# Supplementary material for: Promoterless Transposon Mutagenesis Drives Solid Cancers via Tumor Suppressor Inactivation
Source: Cancers (Basel). 2021 Jan 9;13(2):225. doi: 10.3390/cancers13020225 (PMC7827284; doi:10.3390/cancers13020225)
Supplement: Supplementary file 1 [file cancers-13-00225-s001.zip › cancers-1051638-supple-figs.pdf]

Supplementary Materials

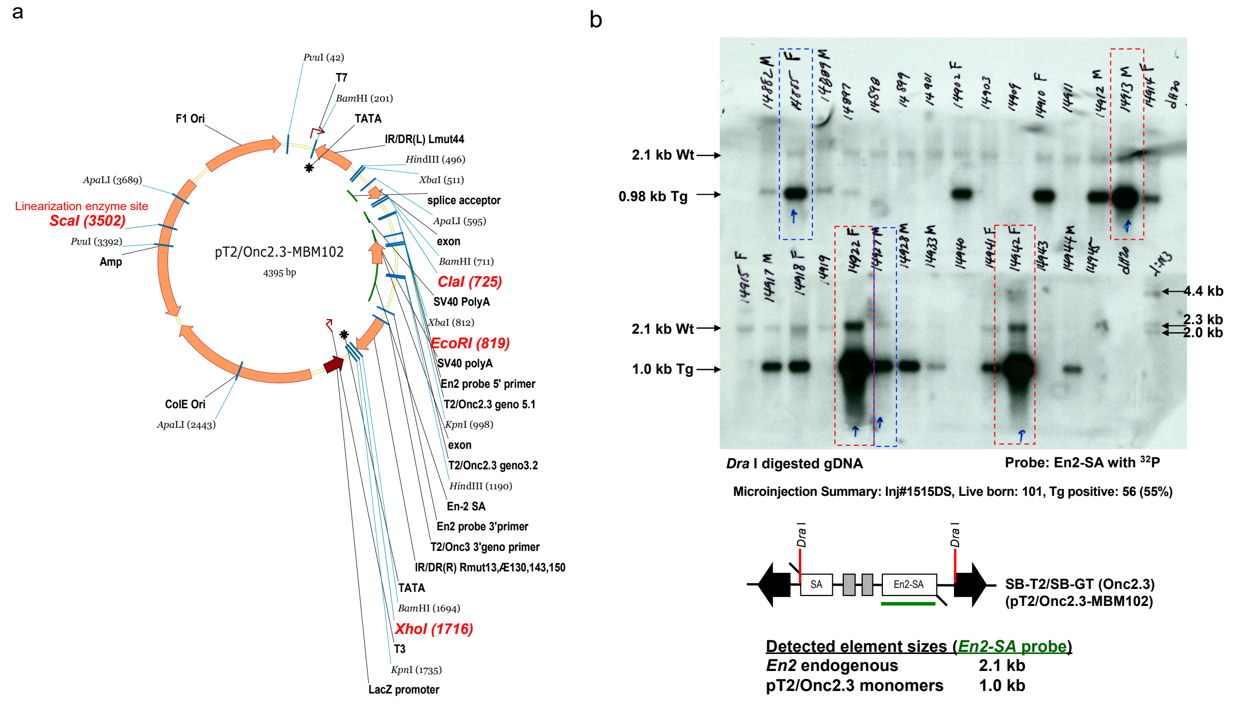

Supplementary Figure S1: Transgenic SB-T2/Onc2.3 founder mice. (**a**) Plasmid map of pT2/Onc2.3-MBM102, denoting essential features and base pair position in parenthesis, used to create the T2/Onc2.3 high-copy transgenic lines. (**b**) Southern blot of T2/Onc2.3 founder mice using a probe corresponding to the *En2*-SA (*Engrailed 2* splice acceptor) element within the SB-T2/SB-GT allele construct that also cross hybridizes to the endogenous *En2* mouse gene on chromosome 5. Genomic DNA digested to completion with *Dra*I restriction enzyme and detected with a radioisotope-^{32}P labeled *En2*-SA probe identifies the diploid endogenous *En2* locus as a discrete 2.1 kb band and any all copies of the T2/SB-GT monomer that have been liberated from the their randomly inserted transgenic loci as a discrete 1.0 kb band. Mice with multi-copy concatemer alleles are identified as containing darker staining bands relative to the two copies of the *En2* endogenous locus band. Blue arrows and dotted boxes denote animals selected for germ line breeding — five separate transgenic founders, three females (TG.14885, TG.14922, and TG.14942) and two males (TG.14913 and TG.14927), were selected for mating with wildtype C57BL/6J mice to confirm the germ line transmission of the SB-T/2SB-GT concatemer alleles to progeny. Mice denoted with blue dotted boxes produced transgenic carrier pups with a transgene copy numbers that differed between individuals, suggesting the presence of two or more independent transgenic concatemer integration sites. Mice denoted with red dotted boxes produced transgenic carrier pups with a transgene copy number that did not differ between individuals, suggesting that they resulted from a single transgenic concatemer integration site. Lines established from founders TG.14913, TG.14922, and TG.14942 were expanded and bred for further experiments. *Bam*HI restriction digested *lambda* (ƛ)-phage DNA is provided as a reference, a small amount was added to the radioisotope-^{32}P labeling reaction with the *En2*-SA probe to show detection on the developed Southern blot.

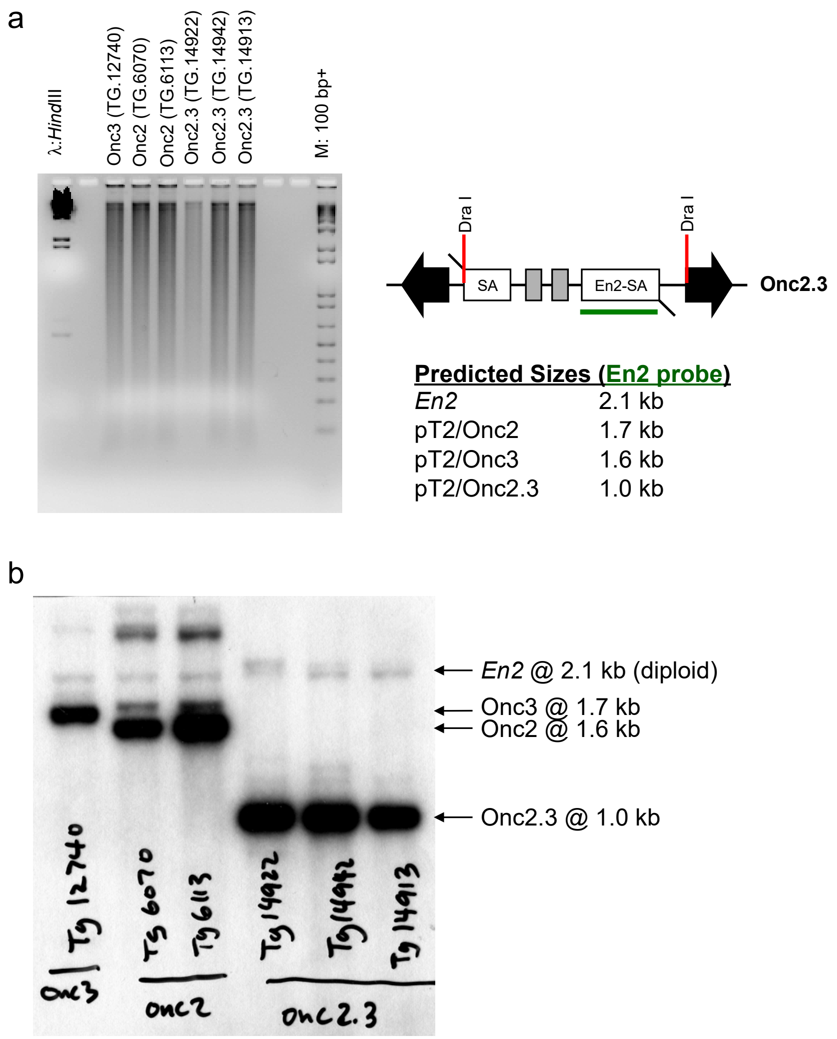

Supplementary Figure S2: Estimating SB transposon concatemer copy number from different SB strains. (**a**) Inverse image of EtBr stained agarose gel (1.2% TBE, run at 30 volts for 30 hours) containing genomic DNA digested with *Dra* I for 16 hours at 37 ºC for Southern blot analysis. Molecular weight markers: left, lambda cut with *Hin*dIII; right, 100 bp ladder. (**b**) Southern blot demonstrating relative concatemer copy number differences between high-copy and low-copy SB transposon alleles in homozygous mice with unmobilized transposons. The *En2* site provides a reference for a single copy, diploid gene.

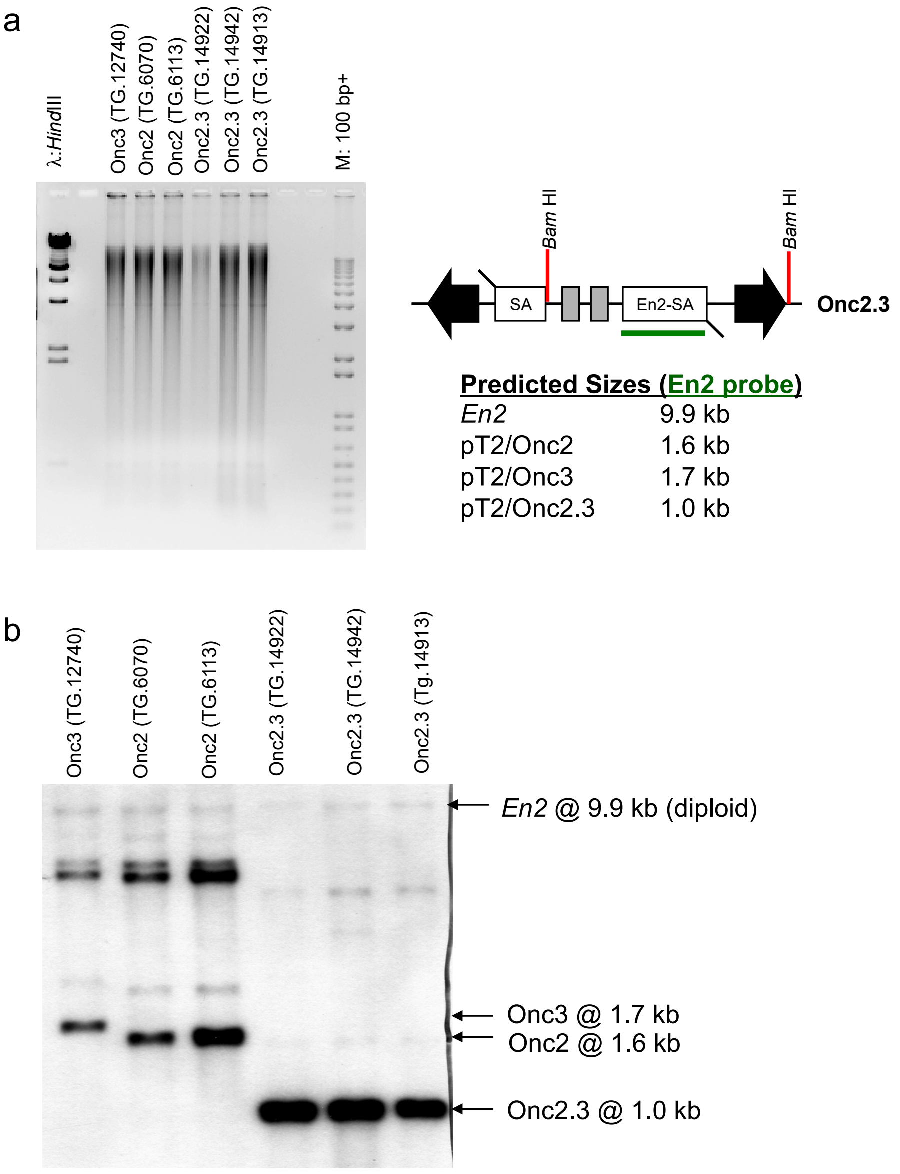

Supplementary Figure S3: Estimating SB transposon concatemer copy number from different SB strains. (**a**) Inverse image of EtBr stained agarose gel (1.2% TBE, run at 30 volts for 30 hours) containing genomic DNA digested with *Bam*HI for 16 hours at 37 ºC for Southern blot analysis. Molecular weight markers: left, lambda cut with *Bam*HI; right, 100 bp ladder. (**b**) Southern blot demonstrating relative concatemer copy number differences between high-copy and low-copy SB transposon alleles in homozygous mice with unmobilized transposons. The *En2* site provides a reference for a single copy gene.

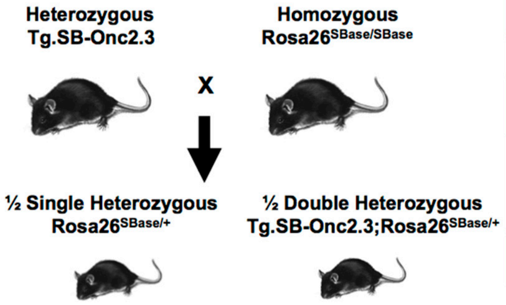

All SB-Onc2.3 alleles combined		
	Observed	Expected
Single Heterozygous controls (Rosa26SBase/+)	148 (78%)	94.5
Double Heterozygous carriers (Tg.SB-Onc2.3; Rosa26SBase/+)	41 (22%)	94.5
Total	189	χ^2=60.557 *P*<0.001

c

SB-Onc2.3 allele	TG.14922		TG.14942		TG.14913	
	Observed	Expected	Observed	Expected	Observed	Expected
Single Heterozygous controls (Rosa26SBase/+)	37 (90%)	20.5	51 (72%)	35.5	60 (78%)	38.5
Double Heterozygous carriers (Tg.SB-Onc2.3; Rosa26SBase/+)	4 (10%)	20.5	20 (28%)	35.5	17 (22%)	38.5
Total	41	χ^2=25.561 *P*<0.001	71	χ^2=13.535 *P*<0.001	77	χ^2=24.013 *P*<0.001

d

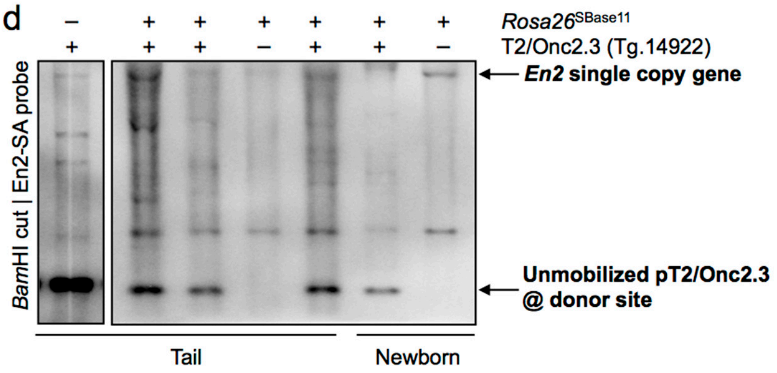

e

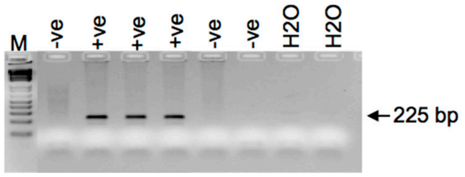

Supplementary Figure S4: Embryonic lethality in double heterozygous mice. (**a**) Mouse cross to generate singe allele control and double allele experimental mice. (**b**) Less than expected number of progeny resulted from matings between double heterozygous carrier mice resulting from crosses between SB T2/Onc2.3 and constitutive SBase expression. (**c**) Onc2.3 (Tg/+); SB'B' (neo/+) double hets are not born in the expected Mendelian frequencies, Goodness of Fit test values provided. (**d**) SB transposition and genome mobilization of T2/Onc2.3 allele *in vivo* by constitutive SBase expression in compound double heterozygous carriers. Southern blot demonstrating change in T2/Onc2.3 (Tg.14922) allele donor site concatemer copy number of in single carrier mice with unmobilized transposons (left lane) and significant depletion in double carrier mice from tail biopsies of wean pups or from whole body newborn kidney specimen. (**e**) Modified SB excision PCR[11] demonstrating transposition of the T2/Onc2.3 (Tg.14922) allele *in vivo* from routine tail biopsies taken from weaned mice for genotyping. Presence of the SB excision PCR product infers SB transposon and SB transposase alleles are all present within the germ line of the weaned mouse.

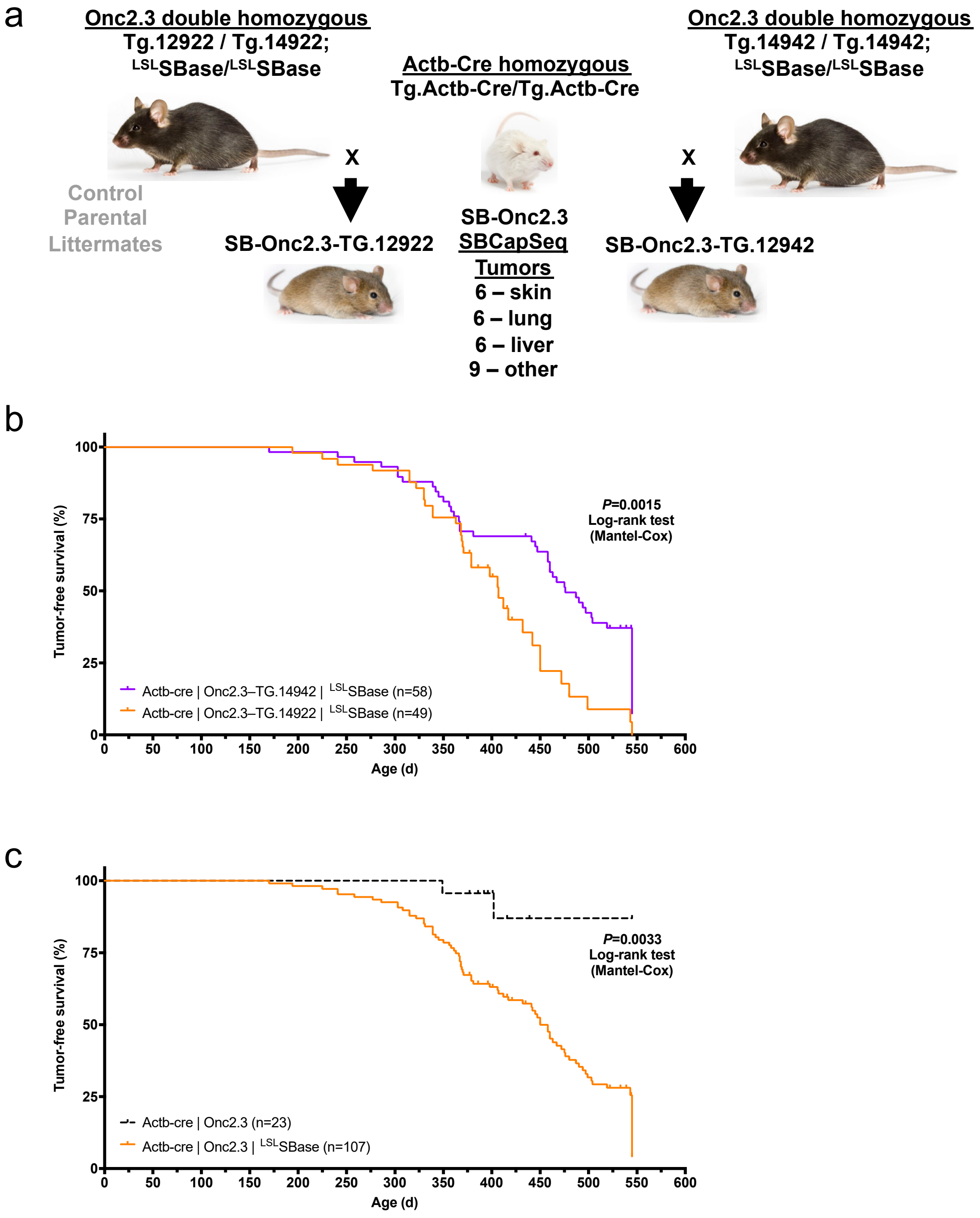

Supplementary Figure S5: Overview of SB-induced tumor studies. (**a**) SB-Onc2.3 breeding strategy and genotype cohorts aged to generate the SB-Onc2.3 experimental and control cohorts of mice used in this study. (**b**) Kaplan-Meier survival plots comparing SB-Onc2.3 cohorts (Mantel-Cox log-rank test, $P = 0.0015$). (**c**) Kaplan-Meier survival plots comparing the combined SB-Onc2.3 experimental and control cohorts (Mantel-Cox log-rank test, $P = 0.0033$).

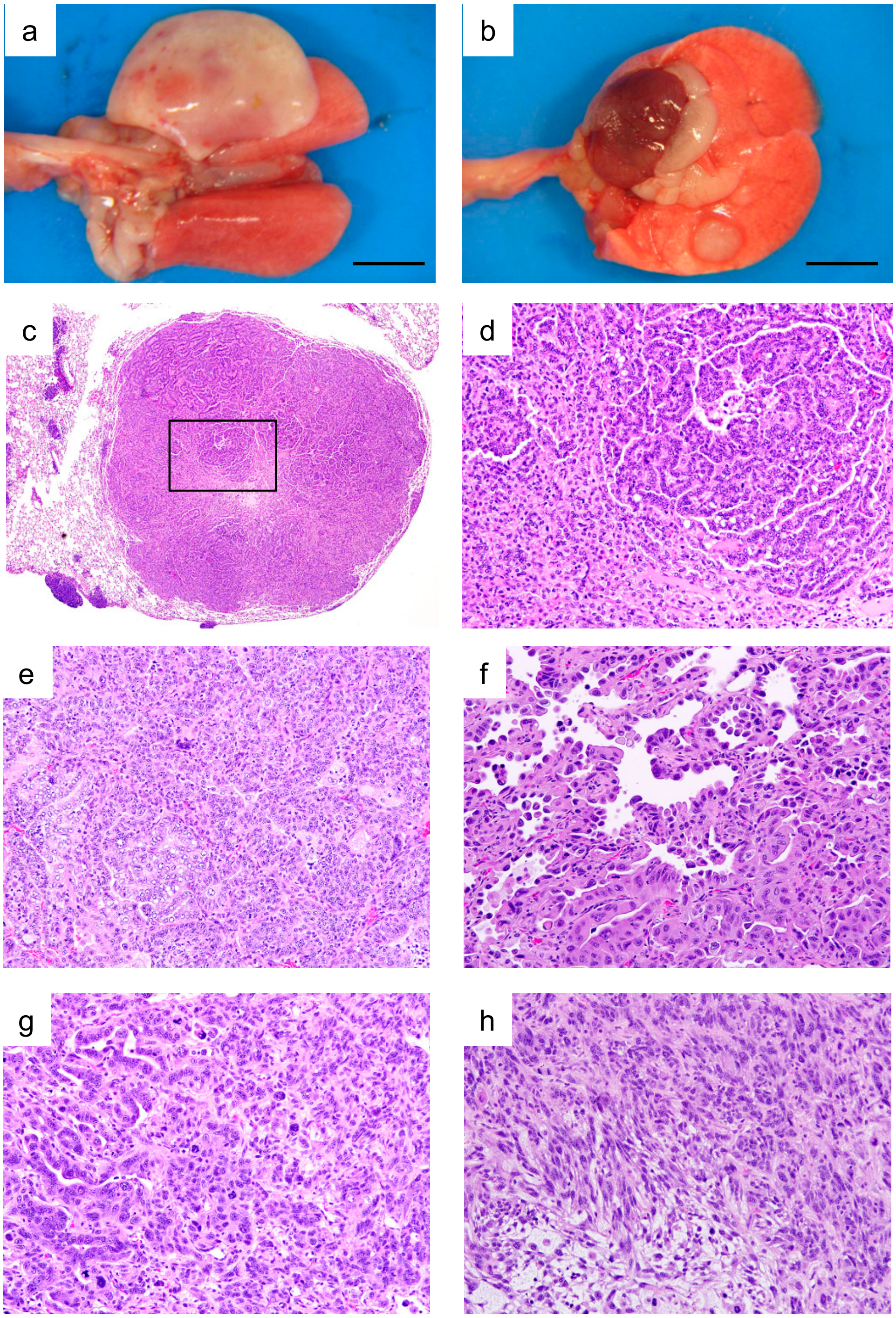

Supplementary Figure S6: Necropsy and histological analysis of SB-Onc2.3 tumors. (**a-b**) Necropsy image of lung tumors: (**a**) white mass (adenocarcinoma) from right apical lobe and (**b**) small white nodule at bottom (adenoma) and at top of lung - large white mass with red area (adenocarcinoma) from the right ventral lobe. Histology and tumor classification from sections of lung masses stained with hematoxylin and eosin (H&E): (**c-d**) Lung adenoma (40×), with inset showing carcinoma differentiation in (**d**) (200×) and Lung (**e-h**) adenocarcinoma areas 200X, (**e**) carcinoma (200×), (**f**) adenocarcinoma (200×), and (**g**) sarcoma (200×). Source image files and additional histology images [79].

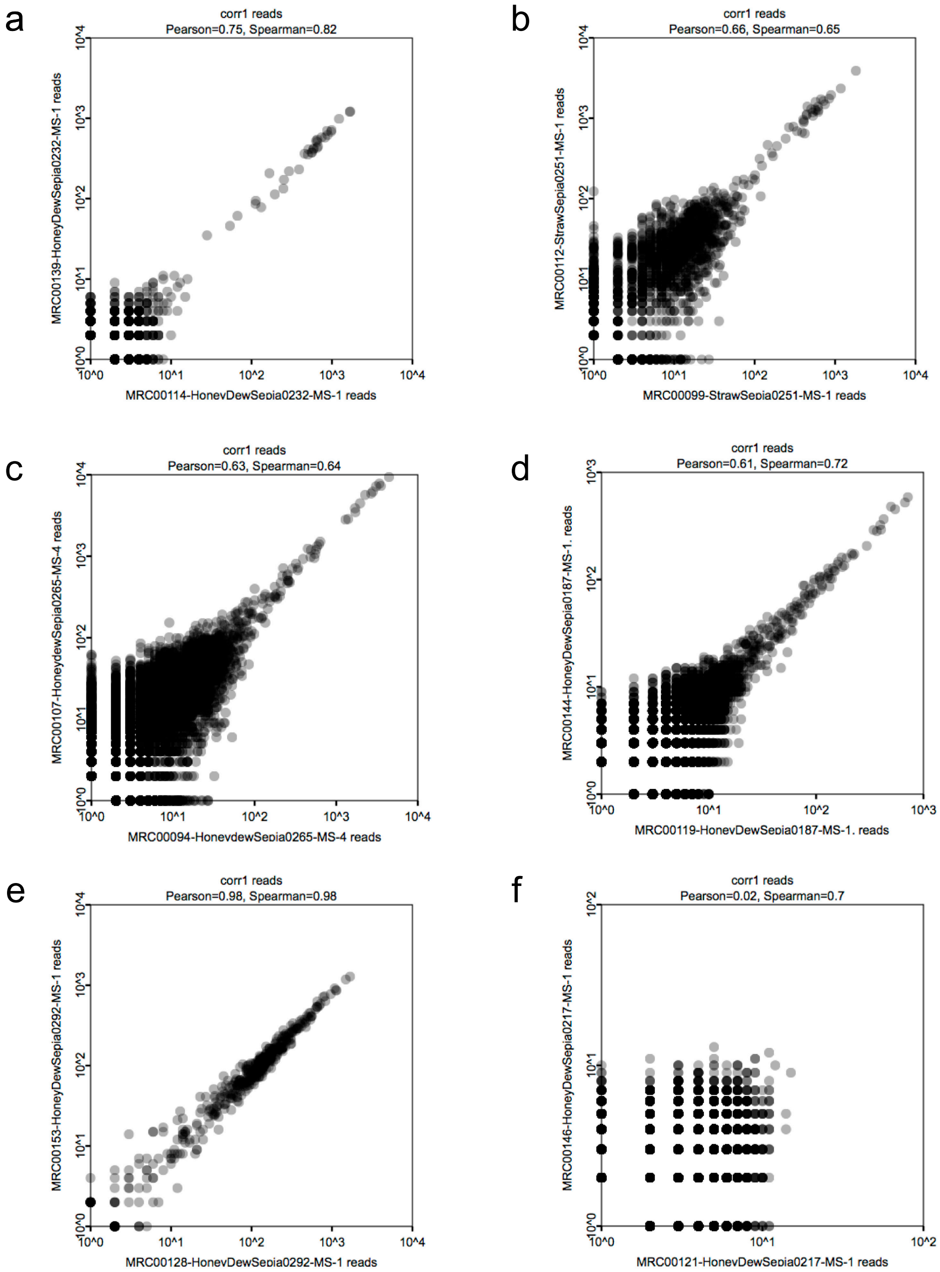

Supplementary Figure S7: Evaluating the reproducibility of SBCapSeq from bulk SB-Onc2.3 tumors. Representative plots of the individual SB insertion sites based on read depth from (**a**) cuSCC, (**b**) HCA, (**c**) LUAA, (**d**) Ovarian adenocarcinoma, (**e**) Schwannoma, and (**f**) astrocytoma genomes from genomic DNAs created from bulk tumor specimens from technical replicate libraries (independent library sequencing runs applied to the same biological specimen isolate) comparing library run 1 (*x*-axis) to library run 2 (*y*-axis). Biological reproducibility of the cuSCC, HCA, LUAA, ovarian adenocarcinoma, and Schwannoma specimen libraries is high, supported by both Pearson's and Spearman's correlation metrics, and identifies the all of SB insertion sites with read depths of 20 or higher (**a-e**). In contrast, technical reproducibility of the astrocytoma specimen libraries is exceptionally low and fails to identify any read depth insertion sites higher than 22 reads (**e**), indicating that the insertions are likely background passenger insertion events that are not clonally selected during astrocytoma progression.

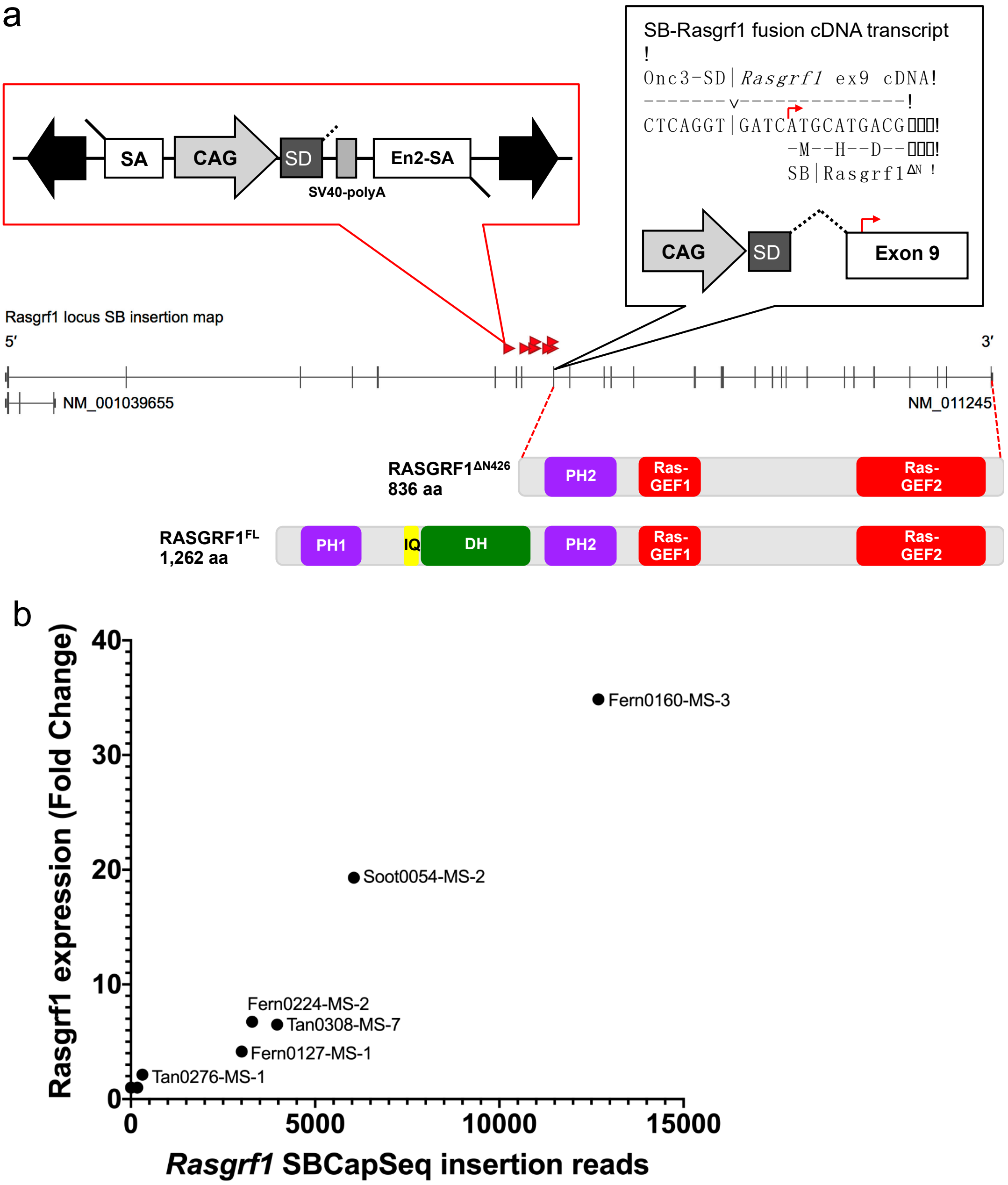

Supplementary Figure S8: Activating SB insertions into the *Rasgrf1* locus drive overexpression an oncogenic delta-N isoform. (**a**) Recurrent activating SB insertions in Onc3-induced lung cancer genomes drives expression of an N truncated RASGRF1$^{\Delta N426}$ isoform that lacks the first 426 amino acids in the full length RASGRF1 protein. The protein domains for RASGRF1 from UniProt (http://www.uniprot.org/uniprot/P27671): PH1 (aa 22 – 130 in the full length), IQ (aa 208 – 233 in the full length), and DH (aa 244 – 430 in the full length) absent in the predicted RASGRF1$^{\Delta N426}$ oncoprotein; PH2 (aa 460 – 588 in the full length), Ras-GEF1 (aa 635 – 749 in the full length) and Ras-GEF2 (aa 1027 – 1259 in the full length) present in the predicted RASGRF1$^{\Delta N426}$ oncoprotein. (**b**) Expression of the delta-N *RASGRF1* is positively correlated with SBCapSeq read depth (Pearson r=0.9761; Spearman r=0.9848; *P*<0.0001; **Supplementary Table 24**).

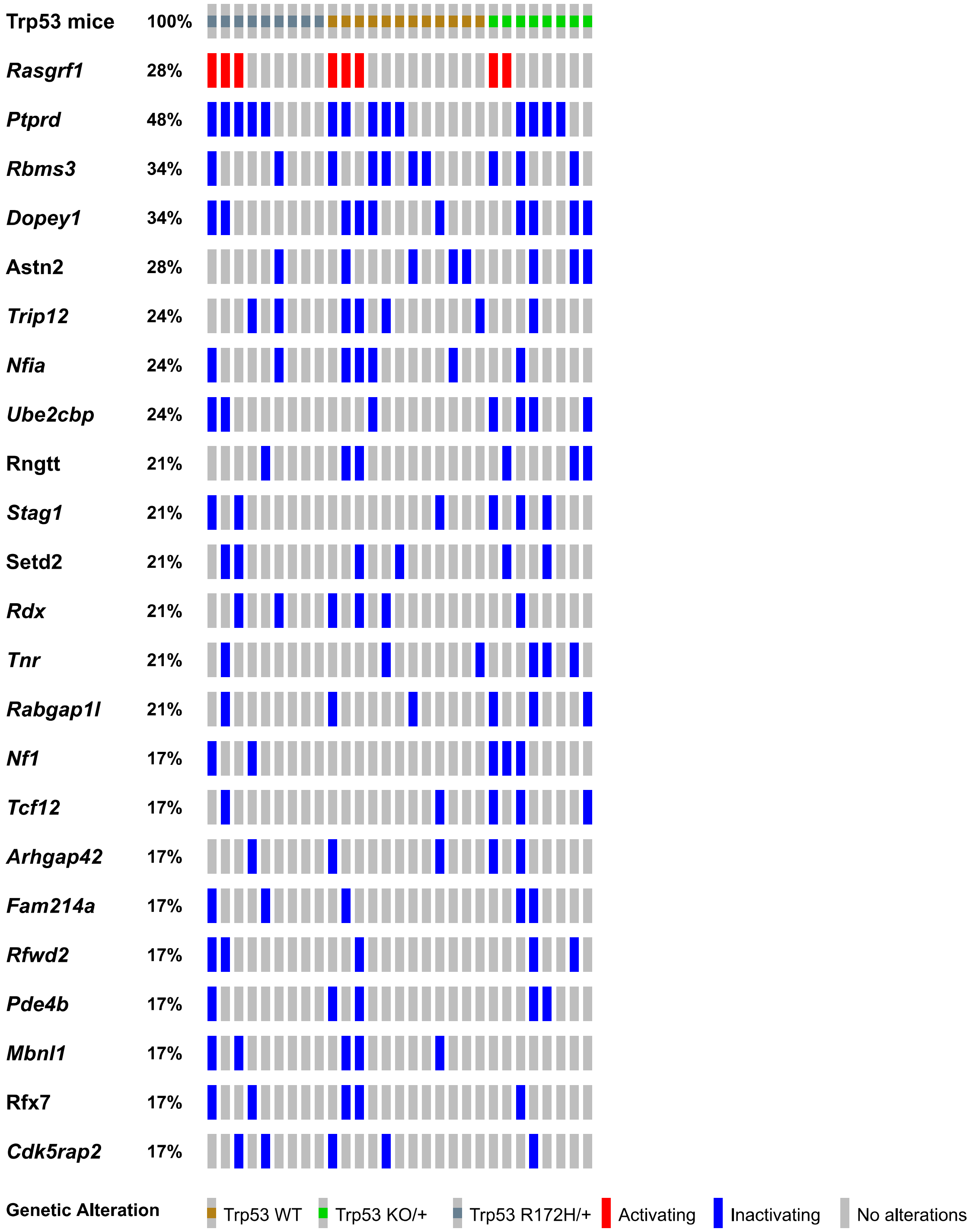

Supplementary Figure S9: Landscape of candidate trunk drivers mutated in SB-induced LUAA with Splink_454T sequencing. SB Driver Analysis applied to Splink_454 SB insertion data (**Supplementary Table 8**) with read depth cutoff of 6 on 29 late-stage HCA genomes. FWER-corrected significant driver genes (rows) were sorted by occurrence within the cohort genomes (columns).

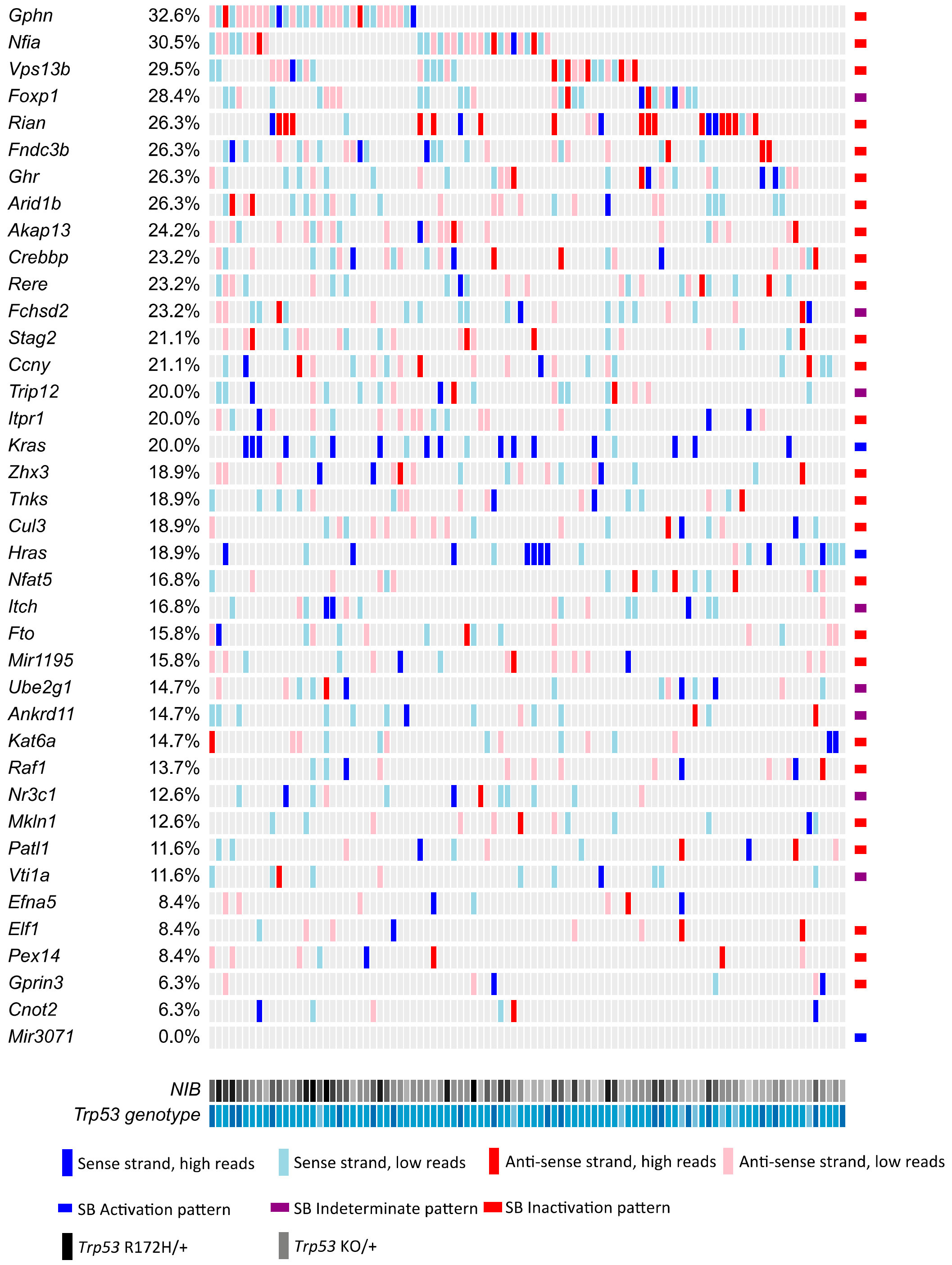

Supplementary Figure S10: Landscape of candidate trunk drivers mutated in SB-induced HCA with Splink_454T sequencing. SB Driver Analysis applied to Splink_454 SB insertion data (**Supplementary Tables 10-12**) with read depth cutoff of 6 on 95 late-stage HCA genomes. FWER-corrected significant driver genes (rows) were sorted by occurrence within the cohort genomes (columns). Waterfall plots were generated on all insertions, and insertion patterns determined by progression driver analysis are shown to the side. NIB, normalized insertion burden from lowest (light gray) to highest (black). *Trp53* genotype shown from *Trp53* $^{+/+}$ (light blue), *Trp53* $^{R172H/+}$ (blue) and *Trp53* $^{KO/+}$ (dark blue).
