# Supplementary material for: Promoterless Transposon Mutagenesis Drives Solid Cancers via Tumor Suppressor Inactivation
Source: Cancers (Basel). 2021 Jan 9;13(2):225. doi: 10.3390/cancers13020225 (PMC7827284; doi:10.3390/cancers13020225)
Supplement: Supplementary file 1 [file cancers-13-00225-s001.zip › cancers-1051638-supple-note.pdf]

Supplementary Notes S1–S3 for manuscript: Promoterless Transposon Mutagenesis Drives Solid Cancers via Tumor Suppressor Inactivation. 2020. Aziz Aiderus, Ana M. Contreras-Sandoval, Amanda L. Meshey, Justin Y. Newberg, Jerrold M. Ward, Deborah Swing, Neal G. Copeland, Nancy A. Jenkins, Karen M. Mann[1], and Michael B. Mann[1].

Correspondence to K.M.M. at Karen.Mann@moffitt.org and/or M.B.M. at Michael.Mann@moffitt.org

S1. Sequencing of SB insertion sites and SB Driver Analysis from astrocytoma specimens

We observed astrocytomas from both Onc2.3 alleles (**Supplementary Table 1**), suggesting the mechanism are not likely to result from the SB concatemer donor site, *per se*. Initially, we were excited to study the astrocytoma masses that occurred frequently in SB-Onc2.3 mice. However, given that the only astrocytoma that was large enough to have a frozen section collected at necropsy (described above) turned out to not have clonal SB insertions, we decided not to pursue the astrocytoma phenotype any further from this experiment. Most of the recovered masses that were ultimately histologically classified as astrocytoma, were small very small and not observable during necropsy. Identification was instead by histological analysis of routine tissue sections from FFPE tissues collected from otherwise healthy tissues. Unfortunately, the yield of high-quality genomic DNA from FFPE samples was not achieved. Thus, our results did not justify proceeding with SBCapSeq analysis for the rest of the astrocytoma samples.

S.2 SB-Onc3 lung cancer initiation and progression in wild type and Trp53 mutant mice

Previous work showed that in wild type mice, SB is able to induce skin squamous cell carcinoma (SCC) and liver hepatocellular carcinoma (HCC) tumors within one year of age (Dupuy *et al*. 2009; http://cancerres.aacrjournals.org/content/69/20/8150.long). To extend these studies we activated an inducible *floxed* allele of the SB transposase (Rosa26-LSL-SB11) using ACTIN-BETA Cre (ACTß-Cre, FVB/N-Tg(ACTB-cre)2Mrt/J; http://jaxmice.jax.org/strain/003376.html) to drive whole-body SB insertional mutagenesis in either a *Trp53* mutant (*Trp53*$^{mut/+}$) or *Trp53* wild type (*Trp53*$^{+/+}$) background. Using a two-generation mating scheme, we first introduced ACTß-Cre onto the background of an inducible *Trp53* mutant strain, either *Trp53*$^{flox/+}$ (FVB.129P2-Trp53tm1Brn/Nci;

http://mouse.ncifcrf.gov/available_details.asp?ID=01XC2) or *Trp53*$^{LSL\text{-}R172H/+}$ (129S4-Trp53tm2Tyj/Nci; http://mouse.ncifcrf.gov/available_details.asp?ID=01XM2) to activate the mutant *Trp53* allele in all cells. We then crossed the resulting compound heterozygous progeny (*Trp53*$^{KO/+}$ or *Trp53*$^{R172H/+}$) and ACTß-Cre (see Figure) onto the background of compound homozygous SB strains containing a low-copy transposon donor concatemer (T2Onc3 (TG.12740) and the inducible SB transposase (Rosa26-LSL-SBase or SBase^{LSL}) to generate the following cohorts: *Trp53*$^{+/+}$*; T2Onc3/+; SBase*^{LSL} and *Trp53*$^{mut/+}$*; T2Onc3/+; SBase*^{LSL} with or without ACTß-Cre. No embryonic lethality was observed in any of the cohorts, hereafter referred to as SB|P53 KO, SB|P53 R172H or SB|P53 WT, as the expected numbers of mice were obtained. We generated and aged a cohort of 202 triple and quadutple-transgenic mice, along with 26 *Trp53*$^{LSL\text{-}R172H/+}$ single-transgenic littermate controls. All cohorts developed skin squamous cell carcinoma (SCC), liver hepatocellular adenoma (HCA), lung alveolar adenoma/adenocarcinoma tumors (LUAA) and other tumors within 16 months of age. Tumor free survival of mice from the SB|P53KO and SB|P53R172H mutant cohorts were statistically significantly reduced compared to SB|P53WT, SB only, or P53$^{R172H/+}$ control cohorts (Kaplan-Meier Survival Log-Rank Test, P<0.0001). No statistically significantly differences in tumor-free survival were observed between SB|P53KO vs. SB|P53R172H (Log-Rank Test, P=0.1832). Additionally, tumor-free survival for the SB|P53WT cohort with inducible SB using ACTß-Cre was not statistically significantly different from that of the published study with constitutive SB (Log-Rank Test, P=0.3406, data not shown). Regardless of *Trp53* genotype, all mice developed at least one tumor during the study, including 10 mice that presented at necropsy with grossly visible masses on one or more lobes of the lung and for which flash frozen tumor tissue was collected. Histological analysis of these masses by a veterinary pathologist with routine H&E sections revealed alveolar adenocarcinoma. Histologically screened H&E stained sections of lungs by a from all remaining animals in the aged cohort for evidence of lung tumors that were not grossly visible at necropsy and identified 18 additional mice with small early stage alveolar adenoma or small but advanced alveolar adenocarcinoma. In total, twenty-eight (13.9% of 202) mice (12 females and 16 males) developed primary tumors in the lung, including 12, 8, and 8 from SB|P53 WT, SB|P53 KO, and SB|P53 R172H, respectively. Formalin-fixed, paraffin embedded (FFPE) lung tumor

specimens were retrieved from paraffin blocks were collected with guidance from a veterinary pathologist to ensure tumor purity remained high.

S3. Sequencing of SB insertion sites and Common Integration (CIS) Analysis from SB|P53 Lung Masses

We used 10 flash frozen and 18 FFPE lung tumor specimens collected at necropsy to create genomic DNA and sequenced using the 454-Splink method to sequencing SB insertion events. For FFPE specimens, xylene was used to remove paraffin and genomic DNA was quantified and subjected to whole genome amplification (WGA) to obtain sufficient quantities for splinkerette-mediated PCR and high-throughput sequencing using the Roche 454 Titanium platform. This method, SB Driver Analysis, considers all uniquely mappable TA dinucleotides in the coding region of each RefSeq gene as a fraction of all uniquely mappable TA dinucleotides in the mouse genome. The population frequency of transposon insertions in each Refseq gene is then calculated (after filtering out insertions on transposon donor chromosomes). Chi-square analysis then calculates the probability of the transposon mutating a TA in a given gene with a higher frequency than predicted by chance. We first analyzed the data without promoter sequence (0 kb), and then reanalyzed the data after including 15 kb of promoter sequence. Four related SB Driver Analyses were performed based on *Trp53* genotype: 1) SB|P53 ALL includes SB insertions from all mice with sequenced lung tumors, regardless of *Trp53* genotype were combined together; 2) SB|P53 WT includes only *Trp53*$^{+/+}$ mice with lung tumors; 3) SB|P53 KO includes only *Trp53*$^{KO/+}$ mice with lung tumors; and, 4) SB|P53 R172H includes only *Trp53*$^{R172H/+}$ mice with lung tumors.
